# Auditory Processing under Cross-Modal Visual Load Investigated with Simultaneous EEG-fMRI

**DOI:** 10.1371/journal.pone.0052267

**Published:** 2012-12-14

**Authors:** Christina Regenbogen, Maarten De Vos, Stefan Debener, Bruce I. Turetsky, Carolin Mößnang, Andreas Finkelmeyer, Ute Habel, Irene Neuner, Thilo Kellermann

**Affiliations:** 1 Department of Psychiatry, Psychotherapy, and Psychosomatics, Medical School, RWTH Aachen University, Aachen, Germany; 2 JARA Translational Brain Medicine, University of Oldenburg, Oldenburg, Germany; 3 Neuropsychology Laboratory, Department of Psychology, University of Oldenburg, Oldenburg, Germany; 4 Department of Electrical Engineering, ESAT-SCD, KU Leuven, Leuven, Belgium; 5 Department of Neurology, Biomagnetic Center, University Hospital Jena, Jena, Germany; 6 Department of Psychiatry, University of Pennsylvania, School of Medicine, Philadelphia, Pennsylvania, United States of America; 7 Institute of Neuroscience, Newcastle University, England, United Kingdom; University of Rome, Italy

## Abstract

Cognitive task demands in one sensory modality (T1) can have beneficial effects on a secondary task (T2) in a different modality, due to reduced top-down control needed to inhibit the secondary task, as well as crossmodal spread of attention. This contrasts findings of cognitive load compromising a secondary modality’s processing. We manipulated cognitive load within one modality (visual) and studied the consequences of cognitive demands on secondary (auditory) processing. 15 healthy participants underwent a simultaneous EEG-fMRI experiment. Data from 8 participants were obtained outside the scanner for validation purposes. The primary task (T1) was to respond to a visual working memory (WM) task with four conditions, while the secondary task (T2) consisted of an auditory oddball stream, which participants were asked to ignore. The fMRI results revealed fronto-parietal WM network activations in response to T1 task manipulation. This was accompanied by significantly higher reaction times and lower hit rates with increasing task difficulty which confirmed successful manipulation of WM load. Amplitudes of auditory evoked potentials, representing fundamental auditory processing showed a continuous augmentation which demonstrated a systematic relation to cross-modal cognitive load. With increasing WM load, primary auditory cortices were increasingly deactivated while psychophysiological interaction results suggested the emergence of auditory cortices connectivity with visual WM regions. These results suggest differential effects of crossmodal attention on fundamental auditory processing. We suggest a continuous allocation of resources to brain regions processing primary tasks when challenging the central executive under high cognitive load.

## Introduction

The brain’s capacity to re-allocate resources and to deal with its attentional capacities is relevant for survival and serves adaptive functioning [1], [2]. How limited processing resources are managed between sensory modalities which are implicated simultaneously via two or more different tasks is however not fully understood. Cross-modal processing has been subject to several experimental investigations [3], [4], [5]. The results can be subsumed under different theoretical frameworks: The ‘automaticity theory’ [6] states automatic processing to be present in the unattended secondary task and immunity to cross-modal influences [7], [8]. Several studies have found evidence for an absence of crossmodal effects on secondary task processing [9]. In contrast, the ‘gain-load theory’ [1] suggests that the primarily engaged modality uses the limited capacities which causes inhibition and thereby decreased processing of secondary input. This was also supported by others [10] and moderated by the assumption of differential effects on undistractable and distractable components of crossmodal attention (e.g. reduced distraction effect but intact automatic change-detection mechanisms, [11]).

Recently, Haroush and colleagues [12] have reported evidence for yet another alternative. In a perceptually demanding visual attentional blink paradigm healthy young participants showed cross-modal augmentation processing of unattended sounds. This was interpreted as a consequence of executive control due to cognitive overload resulting from the attended task. The decrease in executive control challenged the otherwise effective suppression of irrelevant input [13]. In contrast to the gain-load theory, the effects expected here on secondary task processing are beneficial rather than detrimental.

Another alternative explanation for advantageous crossmodal effects may include generalized attention, a concept attributed to a spread of cognitive alertness [14], [15]. This may be caused by the challenging task in the primary modality, which supports the notion of attention being a general, modality-independent cognitive resource serving beneficial purposes for other modalities.

One family of crossmodal effects are primary visual load effects on secondary auditor processing. Most studies focused on the auditory change effects [16]. Results show inconsistencies regarding the directionality of crossmodal effects. Some report decreased MMN amplitudes in the secondary task [1], [10], others find the opposite [17], [18] and there also exist null-findings on potential crossmodal influences [7], [8], [9]). While the MMN reflects active sensory memory processing [19], the N1 as its prerequisite contributes to encoding the sensory memory trace. It acts out stimulus perception as well as feature-detection mechanisms and represents fundamental auditory processing [20]. However, it was usually not distinguished whether standard or deviant processing was affected and which of the two was responsible for the decrease in auditory change detection [12]. It remained open whether the observable effects would already be present during basic tone processing. SanMiguel and colleagues [11] made an exception to this reporting an effect of visual working memory (WM) on the auditory N1. However, memory load was manipulated only on one level and the directionality of this effect (decreasing/increasing) could not be determined. Haroush and colleagues [12] also reported auditory evoked responses (AEPs), however, they also focused on the MMN and the significance of the effect specifically of N1 or P2 amplitudes could not be evaluated.

The aim of the present study was to investigate how stepwise increases in a four-level visual WM design would influence basic auditory processing. Rather than audiotry change effects we wanted to specifically analyze standard tones, representing sensory encoding for the memory trace which is the prerequisite for further higher-level processes such as the auditory change effect.

We used FMRI in order to assess WM manipulation and concurrently recorded event-related potentials (ERP) to measure auditory processing. Although a simultaneous recording of both modalities is not strictly required, it has several advantages: It enables disentangling modality-specific effects but guarantees inferring direct relations due to the measurement simultaneity and the time-point stable task manipulation of both modalities.

Often-replicated fronto-parietal network activations are well-established fMRI correlates of visual WM [21], [22], [23]. Electrophysiological tone responses are characterized by the auditory N1-P2 vertex potential. These ERP components can also be reliably obtained when recorded in the MR scanner and the potential coupling between both measures is a matter of ongoing research [24].

Our hypotheses were based on a successful manipulation of visual WM load, which would result in uni-modally enhanced fMRI activation patterns in WM-related areas. Cross-modal effects in fundamental auditory processing were investigated via AEPs simultaneously measured, as well as measured outside the scanner in a different sample. Our simultaneous measurement setup would further help us to provide a more refined answer to how the spatial and temporal correlates of potential crossmodal load effects would manifest themselves by reciprocally informing one measurement modality by the results of the other [25], [26], [27].

## Methods

### Ethics Statement

The experimental set-up conformed to the Code of Ethics of the World Medical Association and the study was approved by the ethics board of the medical faculty, RWTH Aachen University. Participants gave written informed consent on the study protocol.

### Subjects

The group of participants consisted of 15 healthy adults (7 females, *M* age = 25.60 yrs., *SD* = 2.87) for simultaneous EEG-fMRI (inside) measurements and 8 healthy adults (5 females, *M* age = 24.25 yrs., *SD* = 3.20) for EEG-only (outside) measurements (5 subjects were measured inside and outside, with 12 months in between measurements,). Participants were recruited through local advertisements, followed by a detailed screening which confirmed a negative history of psychiatric disorder, neurological illness or current substance abuse. All participants were right-handed [28], had normal or corrected-to-normal vision and fulfilled MR scanning inclusion criteria. Two participants were excluded from the EEG analysis and subsequent integration of fMRI and EEG due to low EEG signal quality. This reduced the final sample for EEG and EEG-informed analyses to 13 participants.

### Stimuli and Task

**Figure 1:**
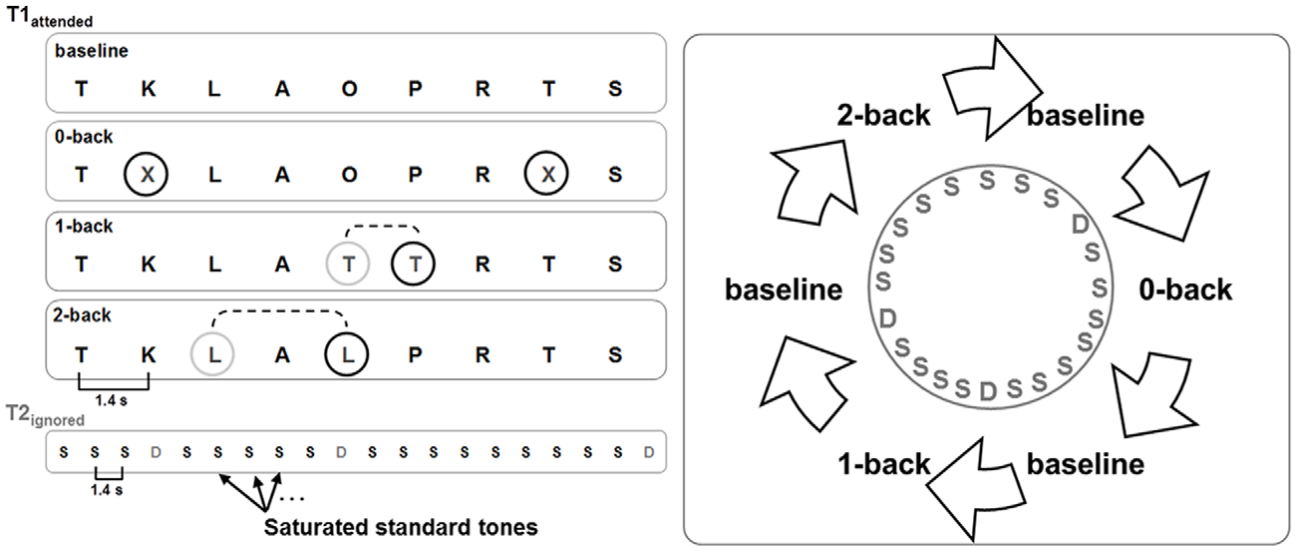
While presenting participants with an attended visual n-back task we investigated cross-modal effects of ignored auditory events consisting of frequent standard tones of 1000 Hz and infrequent deviant tones of 1300 Hz on brain responses (ISI = 1.4 s). The experiment consisted of 5 blocks per condition (27****s) and 15 fixation (baseline) blocks (15****s).

The experiment (Figure 1) consisted of an attended visual n-back task (T1) with four conditions for parametric modulation of WM load. The baseline condition (‘fixation’) included watching a presented series of letters, the ‘0-back’ condition required subjects to respond via a button-press to the target letter ‘X’, in ‘1-back’, they had to respond to two consecutive identical letters, and in ‘2-back’, they had to respond to letters identical to the one presented two trials before. Letters in 1-back and 2-back could be any of the alphabet, except 'X'. Each of the 3 condition-blocks (0-back, 1-back, 2-back) appeared five times, interspersed with 15 baseline blocks. Stimuli were presented for 500 ms every 1.4 s for block duration of 27 s in the n-back conditions and 15 s in the baseline. Every block was initiated by a 3 s task instruction. A fixed order was repeated five times.

The unattended task (T2) consisted of frequent standard tones (1000****Hz, ∼60 dB) and infrequent deviant tones (1300****Hz, ∼60 dB), continuously presented every 1.4 s with a standard oddball ratio of 9∶1. This resulted in approximately 14–16 standards and 1–2 deviants per experimental block (9–11 standards and 1 deviant during fixation) and a total of 360 standards and 45 deviants. Tones and visual stimuli were time-locked to each other: tones were presented first and always followed after 500 ms by visual stimuli. The presentation of the tones was not directly triggered to the MR pulse to prevent from time-locking of the MR artifact signal and the EEG signal. The very first three standards and off-timed tones (exceeding ±30 ms deviance from inter-stimulus-interval) were excluded from further analysis. This was the same for standards following deviants and first standards of each block.

### Procedure

Participants were prepared for the simultaneous measurement and comfortably placed in the MR scanner with the right hand’s index finger placed on a response button (LUMItouch™, Lightwave Technologies, Richmond, Canada). The experimental stimuli were presented using Presentation® (Neurobehavioral Systems Inc., San Francisco, CA). Tones were presented at ∼60 dB via MR-compatible heaphones (Behringer ®).

In order to guarantee that EEG quality of auditory evoked potential was reliable inside the scanner, eight control participants were prepared for an EEG-only measurement and measured in a dimly-lit room, in a supine position, wearing goggles and headphone to ensure comparability of experimental influence.

Subjects were instructed to engage in the visual n-back task and to ignore all tones.

### Data Acquisition and Analysis

In order to investigate cross-modal effects caused by increasing visual WM load on basic auditory processing, we followed a reciprocal analysis strategy of informing the EEG analysis by effects found in the fMRI and vice versa.

### Behavior

Behavioral responses (hit rates and reaction times) were analyzed one-way repeated measures ANOVAs in IBM® SPSS® (version 20). These models included the within-subjects factor ‘n-back’ with three levels (leaving out fixation). Post-hoc tests were performed using paired *t*-tests and Bonferroni correction.

### FMRI Preprocessing and Whole Brain Analysis

fMRI data were obtained on a 3 Tesla Tim Trio® MR scanner (Siemens Medical Systems, Erlangen, Germany) during one run with an echo-planar imaging (EPI) T2* contrast sequence sensitive to blood oxygenation level dependent (BOLD) changes (3.125×3.125×3.4 mm^3^ voxel size, 64×64 matrix, 200×200 mm^2^ FOV, 33 3.4 mm-thick axial (AC-PC) slices with whole brain coverage (0.51 mm gap), ascending acquisition, TR/TE = 2000/30 ms, 76° flip angle [29], 360 volumes). Data analysis was performed using SPM8 (Wellcome Department of Cognitive Neurology, London, UK). Preprocessing of data included realignment of data to correct for head movement, coregistration of the mean EPI scan onto the SPM8 grey matter tissue probability map, and normalization using the unified segmentation approach [30]. Images were resampled to a voxel size of 1.5×1.5×1.5 mm^3^ and smoothed with an isotropic 8 mm FWHM (full width at half maximum) Gaussian kernel.

On a single-subject level, four regressors (one for each WM condition) were created by convolving the respective box-car function with the canonical double-gamma hemodynamic response function (HRF) [31]. Realignment parameters were included as covariates of no interest and the session mean was regressed on a constant term. Prior to parameter estimation, a 128 s high-pass filter was applied. Serial auto-correlations were accounted for by including a first order autoregressive model (AR-1). Simple main contrasts (fixation, 0-back, 1-back, 2-back) were further used for the group-level analysis. A first step was to validate visual WM load by analyzing fMRI activations corresponding to the visual letter presentation. A mixed-effects GLM was used for group-level inference with subjects as random effects and WM conditions as fixed effects. Departures from sphericity were corrected for by variance components assuming a compound symmetry structure for within-subjects (correlated) measures and heteroscedasticity between subjects and conditions.

In order to reveal neural activation corresponding to visual WM load, we carried out an *F*-test, testing for general difference between the four conditions. The statistical parametric map was thresholded at *p*<.05, corrected for multiple comparisons at the voxel-level using Gaussian random field theory (family-wise-error, FWE) and a cluster-extent threshold of 20 voxels. A T-contrast was carried out, testing for a parametric increase of WM-load (conjunction analysis 2-back>1-back ∩ 1-back>0-back ∩ 0-back>fixation). This contrast was thresholded with a combined height and extent threshold technique based on Monte-Carlo (MC) simulations calculated with AlphaSim [32]. Based on an uncorrected threshold of p<.001 and the spatial properties of the residual image an extent threshold of 125 voxels was estimated using 100000 and complied with a family wise error of p<.05.

Activation maxima are reported as MNI-coordinates and anatomical locations are based on the Talairach Client (Lancaster & Fox, Research Imaging Center, University of Texas Health Science Center San Antonio) and the Anatomy Toolbox [33].

### EEG Preprocessing and ERP Analysis

We used a 64-channel MR-compatible EEG system (two BrainAmp MR plus 32-channel amplifiers, BrainProducts GmbH, Gilching, Germany), connected to a MR-compatible electrode cap (Easycap GmbH, Herrsching-Breitbrunn, Germany) with 64 Ag–AgCl electrodes (5 kΩ resistors), 63 of which covered the 10–20 system and one electrocardiogram (ECG) electrode placed ∼10 cm below the left scapula. Electrodes at positions FCz and AFz served as the recording reference and ground electrode, respectively. The online sampling rate was set to 5000 Hz (0.01–250 Hz analog band-pass filter), and electrode impedances were below 20 kΩ. To improve EEG artifact attenuation a sync box (BrainProducts GmbH, Gilching, Germany) was used for optimal synchronization of EEG acquisition with the clock controlling MRI slice acquisition. At the start of each volume acquisition, an event marker was sent to the recording device (Brain Vision Recorder 1.0, BrainProducts GmbH, Gilching, Germany) to enable identification of gradient onsets and to create a template for artifact subtraction.

Offline analysis of EEG data was accomplished using Brain Vision Analyzer software, version 2.0 (BVA 2.0, Brainproducts, Gilching, Germany) and EEGLAB, version 8.0.3.5b [34]. Continuous EEG data underwent gradient artifact removal using the template matching algorithm in BVA [35]. After gradient artifact removal, the data were low-pass-filtered with a digital infinite impulse response filter (IIR, 70 Hz, 48 dB slope) and downsampled to 500 Hz. Cardiac pulse correction was carried out based on an automatically detected pulse template in the ECG channel. Markers were set at highly correlated (>0.7) and above-threshold amplitude (0.4–1.4) time-points. Cardiac pulse markers were visually confirmed and the data subsequently exported to EEGLAB in order to apply a channel-wise optimal basis set procedure [36], [37] as implemented in the EEGLAB-plugin FMRIB 1.2. Data was then re-referenced to linked mastoids (mean TP9-TP10). Independent component analysis (ICA, extended infomax) [38], [39] revealed components relating to eye movement which were removed from the data (maximally two components were removed). Data were filtered (1–30 Hz) and epochs exceeding 125 µV were rejected from further analysis.

Fundamental auditory processing was assessed by analyzing saturated standard tones (Figure 1) which were conceptualized as any tone not appearing as first tones of a block or following a deviant, leaving 372 trials total. Since blocks were uniformly distributed across the experiment a direct comparison of conditions did not include general adaptation effects across time.

Cross-modal effects on event-related auditory potentials (AEPs) were assessed by extracting auditory events in different WM conditions and analyzing condition-specific peak amplitudes and latencies. AEP epochs included 700 ms around the tones (−100 to 600 ms post-stimulus onset, baseline-corrected) and were averaged within each of the four visual WM load condition. Based on other work [1], [8], [9] we extracted peak amplitudes from electrode position Fz. The absolute N1-P2 peak-to-peak values were extracted using an N1 search window between 80–140 ms and a P2 search window between 170–210 ms for each of the four conditions. This yielded four values per subject (fixation, 0-back, 1back, 2-back). N1-P2 amplitudes and the latencies of N1 and P2 peaks were statistically analyzed within generalized linear estimating equations (GEE) in IBM® SPSS® (version 20). The statistical models included main effect of the factors 'n-back' (four levels). Post-hoc tests were performed using paired *t*-tests and Bonferroni correction.

### EEG-informed fMRI Covariate Analysis (ANCOVA)

We carried out three ANCOVA models, each testing for the effect of the mean N1, P2, and N1-P2 amplitude of standards, respectively on a group level. Values (four per subject) were entered into the statistical design as a covariate explaining inter-individual BOLD variance after mean-centering. This approach assumes a correspondence of event-related potentials to neural activity measured via changes in BOLD [24], [40], [41]. While allowing for an interaction between the covariate and the main task, we were interested in two contrasts: A *T*-contrast testing for the average effect of the covariate regressors which would represent a general effect of the auditory response on brain activation, as well as an F-contrast testing for the effect of the covariate interacting with the task effect (differences between WM-load conditions). Both contrasts were masked with the effects of interest of the *F*-contrast testing for unsigned differences between visual WM-conditions of the four BOLD regressors (inclusive mask, thresholded at *p*<.05 uncorrected) and thresholded at *p*<.001 (MC-cluster-corrected, p<.05).

### Region-of-interest Analysis in the Auditory Cortex

Cross-modal effects caused by visual working memory load on basic auditory processing motivated a subsequent individual region-of-interest analysis in primary auditory cortex (AC). Since auditory processing of the tones was not experimentally manipulated (only visual working memory load was), we would have expected null findings in both auditory cortices. However, primary and non-primary AC are the primary generator regions of AEPs [42], [43], [44]. Therefore it was investigated whether the BOLD signal measured in AC complemented the effects of cross-modal manipulation of AEP amplitudes.

For this region-of interest analysis, anatomical masks of left and right Heschl’s gyrus were created using the AAL database [45] in WFU Pickatlas [46], [47]. Using MarsBaR [48], the same single subject analysis was performed as described above for the whole brain analysis (four regressors modeling the BOLD response of each WM load condition) separately for each AC. The condition-wise averaged time-series of AC activation were analyzed in IBM® SPSS® (version 20) using generalized linear models with the within-subject factor condition (four levels).

### Psychophysiological Interaction (PPI)

While the region-of-interest analysis would allow specific insight into the effect of experimental conditions in a specific region it was also of interest how functional connectivity patterns of this region and others would emerge in different WM conditions.

In the subsequent PPI analysis we therefore extracted the individual time-series of left and right primary AC (same masks as used for the ROI analysis). After deconvolution, data vectors were multiplied with the respective box-car functions representing one WM condition each and reconvolved with the canonical HRF [49]. On a single subject level, the data vector (representing one of four conditions) was implemented as a PPI regressor. The convolved main effect of each condition, the seed region’s time-course, and six realignment parameters as well as an intercept were entered as covariates of no interest into the analysis.

After model estimation, parameter estimates of the PPI regressors from each subject’s four first-level analyses were entered into a mixed-effects GLM for group-level inference with subjects as random effects and four PPI regressors as fixed effects. As in the BOLD-GLM described above, departures from sphericity were corrected for by variance components assuming a compound symmetry structure for within-subjects measures and heteroscedasticity between subjects and conditions. Simple main effects, representing task-related connectivity of AC with in each WM-load condition were thresholded at *p*<0.05 (FWE-corrected), the conjunction analysis testing for effects correlating with parametrically increasing visual WM load was thresholded at p<.05 (MC-cluster-corrected).

## Results

### Neural Activation Patterns of Visual WM Load

**Figure 2:**
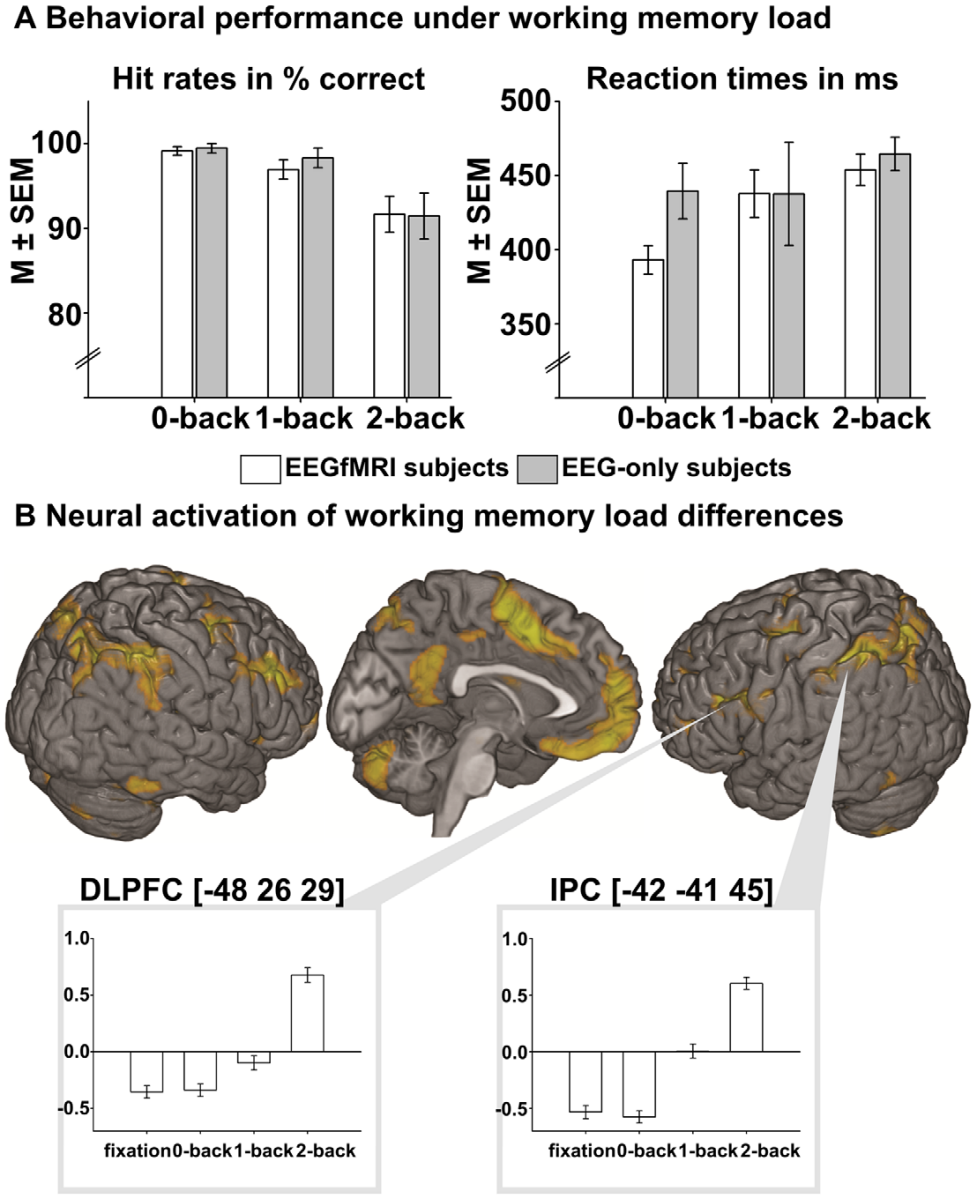
A) Behavioral results of WM-load manipulation on subjects' hit rates (correct %) and reaction times (both *M*±*SEM*). In EEGfMRI subjects (n = 15) GLMs confirmed a significant increase in RTs and decrease in hit rates when WM-load increased. This was replicated in EEG-only subjects (n = 5), but only significant for hit rates. B: Neural activation of visual WM-load, displayed by a contrasts from a random-effects GLM testing for general unsigned differences between visual WM load conditions (*F*-contrast, *F*>14.46, *p*<.05, FWE-corrected, k>20). Within the left dorsolateral prefrontal cortex (DLPFC) and the left inferior parietal cortex (IPC), cluster mean voxel activation (±SEM) were displayed via bar charts. MNI coordinates indicate the location of the maximum within the respective cluster.

The main effect of the WM task (*F*-contrast testing for differences between all conditions, Table 1A, Figure 2) revealed activations in bilateral inferior frontal and prefrontal cortex, supplementary motor area (SMA) and ventromedial prefrontal cortex (vmPFC), middle cingulate gyrus, and precuneus, as well as activations in parietal areas (intraparietal sulcus, inferior parietal lobe and angular gyrus) extending to temporal areas, and several cerebellar clusters.

**Table 1:** Activation patterns corresponding to the effects of visual WM load.

Brainregion	Hemisphere		Clustersize		*T/F*	*p*	x		y	z
**A) Main effect (** ***F*** **-Test)**										
Inferior frontal gyrus	L		2772		32.30	<.001	−35		21	−6
	R		1132		29.21	<.001	30		23	0
Orbitofrontal gyrus	R		408		25.39	<.001	36		54	−6
DLPFC	L		2977		8.47	<.001	−48		26	29
	L		46		2.34	.001	−39		35	−18
	L		27		17.09	.008	−26		17	−21
	R		20		18.15	.004	45		21	12
Middle frontal gyrus	L		143		16.58	.012	−44		47	9
Superior frontal gyrus	L		29		16.91	.009	−30		57	0
SMA	L		14751		8.44	<.001	−5		5	50
vmPFC	L		5385		35.47	<.001	−6		54	−17
	R		45		21.14	.001	9		50	26
Middle cingulate gyrus	L		1352		25.26	<.001	−8		−45	36
	L		56		17.20	.008	−5		−27	45
Precuneus	R		108		18.57	.003	8		−59	26
Inferior parietal gyrus	L		16781		68.32	<.001	−42		−41	45
Angular gyrus	L		118		17.06	.008	−47		−63	26
Insula	L		25		2.30	.001	−47		0	14
	R		166		25.00	<.001	39		−15	17
	R		56		18.80	.003	48		−29	20
Inferior temporal gyrus	L		152		21.42	.001	−54		−63	−15
Fusiform gyrus	L		25		16.95	.009	−32		−36	−18
	R		268		23.36	<.001	57		−56	−17
	R		50		19.70	.001	42		−62	−15
Thalamus	R		617		22.46	<.001	12		−6	−2
Putamen	R		411		2.42	.001	21		11	2
Lobule VIIa crus I	L		2077		45.25	<.001	−32		−65	−32
	R		133		2.98	.001	36		−78	−39
Lobule VI	R		5229		44.42	<.001	35		−51	−32
Lobule VIIIa	R		116		25.36	<.001	39		−44	−54
Lobule VIIa crus II	R		172		19.55	.002	38		−63	−51
Substantia nigra	L		69		18.87	.003	−8		−20	−12
**B) Parametric effect (** ***T*** **-Test conjunction)**										
Precentral gyrus	R	236		4.35		–	30	−3		47
SMA	R	449		4.85		–	5	9		51

A) The *F*-contrast (*F*>14.46) resulted from a group-level GLM, testing for general differences between all visual WM conditions, *p<.05,* FWE-corrected, k>20). B) The *T*-contrast conjunction (*T*>3.24) combined the effects of 2-back>1-back ∩ 1-back>0-back ∩ 0-back>fixation, *p*<.05, MC-corrected, k>125). P-values are only reported if surviving voxel-level FWE correction. DLPFC = dorsolateral prefrontal cortex, SMA = supplementary motor area, vmPFC = ventromedial PFC.

The parametric effect of WM load (*T*-contrast conjunction 2-back>1-back ∩ 1-back>0-back ∩ 0-back>fixation, Table 1B) showed activation in Area 6 (precentral gyrus) and supplementary premotor area (SMA).

### Behavioral Effects of Visual WM Load

The GLM analyzing participants’ hit rates and reaction times each showed a significant main-effect of 'n-back' (hit rates: Wald *χ^2^*(2) = 17.74, *p*<.001; RTs: Wald *χ^2^*(2) = 35.47, *p*<.001). Post-hoc tests of hit rates showed a significant decrease from condition 0-back to 2-back (*t*(14) = 3.92, *p*<.001) and from condition 1-back to 2-back (*t*(14) = 3.84, *p = *.013). Post-hoc tests of RTs showed a significant increase condition 0-back to conditions 1-back (*t*(14) = −4.11) and 2-back (*t*(14) = −4.10) (Table 2 and Figure 2).

**Table 2:** Mean values of subjects’ behavioral performance (mean percent correct hit rates and reaction times in ms, *SD* in brackets) in three visual WM conditions.

	Hit rates		Reaction times	
WM-condition	EEGfMRI	EEG-only	EEGfMRI	EEG-only
**0-back**	99.24 (1.70)	99.43 (1.28)	391.38 (33.25)	439.56 (41.82)
**1-back**	97.33 (3.96)	98.29 (2.55)	429.13 (58.33)	437.57 (77.43)
**2-back**	92.00 (7.26)	91.43 (2.71)	445.38 (42.48)	464.43 (25.06)

‘EEGfMRI’ refers to the participants measured with simultaneous EEG-fMRI (n = 15), ‘outside’ refers to controls measured outside the scanner. Due to recording problems, behavioral data were unavailable for three outside participants which decreased the sample size to n = 5.

In control participants (EEG-only), hit rates also significantly increased (main effect of ‘n-back’, Wald *χ^2^*(2) = 16.09, *p*<.001). Post-hoc tests showed a significant decrease in hit rates from condition 0-back to 2-back (*t*(4) = 3.50, *p*<.001) and from condition 1-back to 2-back (*t*(4) = 2.73, *p = *.006). Although on a descriptive level, the effects were comparable to those observed in inside data the main effect of ‘n-back’ on RTs was not significant (Wald *χ^2^(2) = 3.95, p<.139*).

### Cross-modal Effects of Visual WM-load on AEPs

Generally, EEG data quality was similar after correction of MR- and CB-artifacts of EEG data measured inside the scanner, and signal-to-noise ratios ('noise' being defined as the difference of an odd-even split) were not significantly different between EEG-fMRI and EEG-only data (t(21) = −1.26, p = .13). This supports former reports of valid data recorded from simultaneous continuous measurements compared to interleaved gap measurements [50] or measurements inside the MR scanner without applying HF pulses [51].

**Figure 3:**
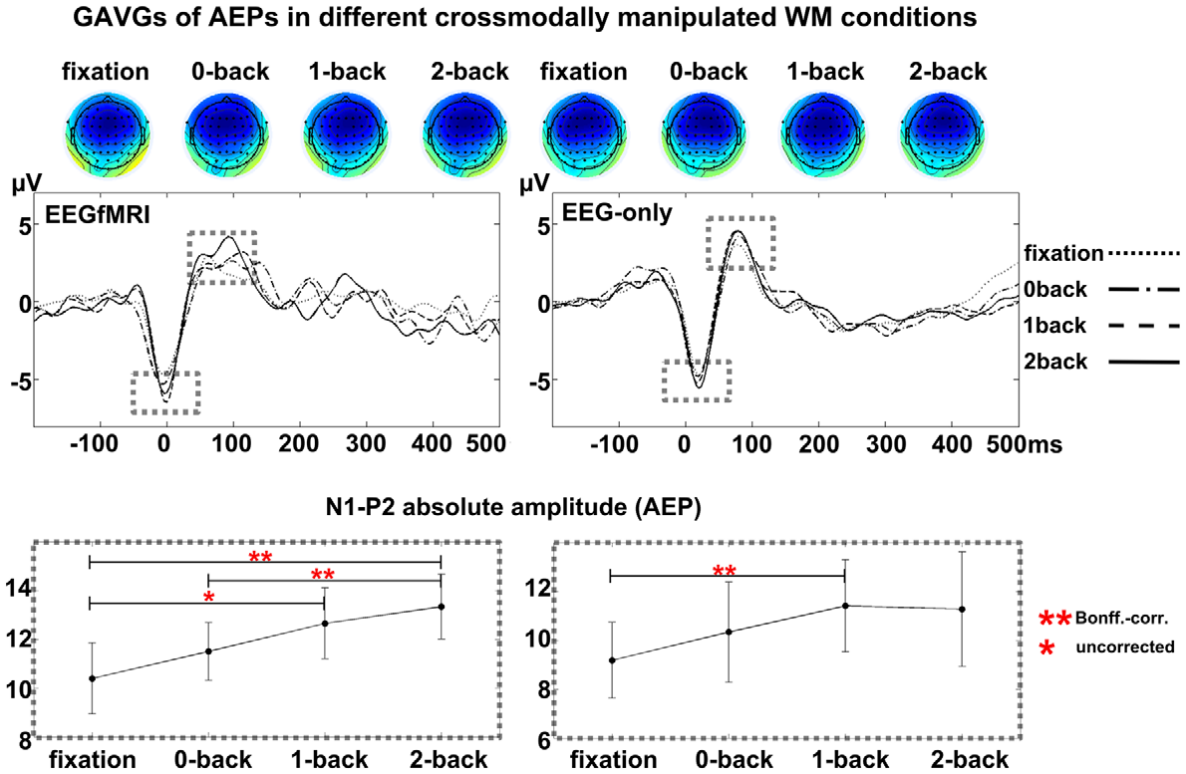
Cross-modal effects of visual WM load on auditory processing. Grand average waveforms represent the evoked responses to unattended standard sounds, measured inside the scanner (EEG-fMRI), and outside (EEG-only) under different crossmodal visual WM-load manipulations. For each WM condition, topographic maps are shown at the latency of the N1 peak at Fz. Line plots below the figures show the condition-effect on AEPs (absolute peak-to-peak N1-P2 amplitude).

N1-P2 amplitude of standard tones of EEG data from inside the scanner showed a significant main effect of ‘n-back’ (Table 3, Figure 3; Wald *χ^2^*(3) = 15.66, *p*<.001). Post-hoc pairwise comparisons showed that with increasing WM load, AEP amplitudes continuously increased from fixation to all subsequent WM-conditions. The difference was significant (Bonferroni-corrected for all possible comparisons) between fixation and 2-back (*t*(12) = −2.62, *p* = .002) as well as between 0-back and 2-back (*t*(12) = −3.03, *p* = .01). The comparison between fixation and 1-back (*t*(12) = −2.41, *p* = .03) was only significant if not Bonferroni-corrected and the other comparisons (fixation compared to 0-back, 0-back compared to 1-back and 1-back compared to 2-back) were not significant.

**Table 3:** Auditory evoked potential (absolute N1-P2 amplitude, *M* µV, *SD* in brackets) in different cross-modal visual WM conditions in two different populations.

n-back condition	Standard tones N1-P2 amplitude (*M* µV, *SD)*
**EEGfMRI data**	
Fixation	10.40 (5.23)
0-back	11.50 (4.27)
1-back	12.65 (5.24)
2-back	13.34 (4.79)
**EEG-only data**	
Fixation	9.19 (4.39)
0-back	10.34 (5.80)
1-back	11.41 (5.32)
2-back	11.28 (6.62)

In EEG-only data, crossmodal effects on auditory processing replicated the effects of the data recorded inside the scanner. We found a significant main effect of ‘n-back’ on AEPs (Wald *χ^2^*(3) = 9.49, *p*<.023). Post-hoc tests showed a significant increase from fixation to 1-back (*t*(7) = −2.83, p = .025). No other post-hoc test showed significant differences between conditions.

No significant main effect of ‘n-back’ was found for N1 or P2 latency in EEGfMRI or EEG-only data.

### EEG-informed fMRI Covariate Analysis (ANCOVA)

**Figure 4:**
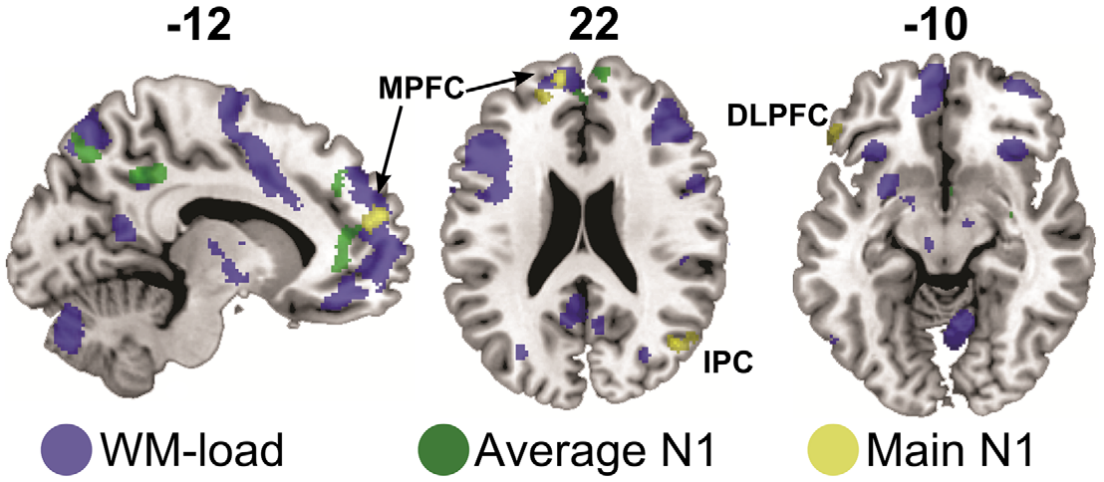
Sagittal and axial slices displaying overlap of inter-subject variations in N1 peak values explaining BOLD variance with frontal core WM regions. Activation patterns resulted from a random-effects GLM including condition-wise N1 peak values for each participant as covariates, inclusively masked (*p*<.05 uncorrected) with the effects of interest of the HRF regressors. Green indicates the average effect of the AEP amplitudes (*F*>6.48, p<.05, MC-corrected), yellow indicates the main effect of AEP amplitudes, accounting for the cross-modal visual WM-condition (*T*>3.29, p<.05, MC-corrected). Both contrasts are overlaid on background blue coloring indicating the main n-back effect shown in Figure 3. DLPFC = dorsolateral prefrontal cortex, MPG = medial prefrontal gyrus, IPC = inferior parietal cortex.

Generally, the N1 peak values of standard tones explained inter-individual subject variance of the BOLD signal in bilateral anterior and posterior cingulate cortex, in left inferior and middle frontal gyrus, as well as inferior temporal gyrus and superior parietal lobe. Subcortical activation was located in the amygdala, caudate nucleus, and hippocampus (*T*-contrast testing for the average effect of the covariate, Figure 4, Table 4).

**Table 4:** N1-peak amplitudes covarying with BOLD signal (ANCOVA).

Brainregion	Hemisphere	Size	T/F	P	x	y	Z
**Average effect N1 (** ***T*** **-Test)**							
Area 6	L	153	4.69		−24	11	53
	L	224	4.91		−27	−12	65
ACC	L	1870	5.77	<.001	−8	36	9
PCC	L	2104	5.60	<.001	−5	−50	41
Middle Frontal gyrus	L	160	5.55		−26	45	6
Inferior temporal gyrus	L	240	6.20		−62	−15	−32
Superior parietal lobe	L	399	4.47	0.017	−11	−69	48
Amygdala	R	387	4.60	0.020	36	−3	5
Caudate nucleus	R	400	4.58	0.017	9	−6	−3
Hippocampus	L	391	6.63	0.019	−29	−29	−20
**Main effect N1 (** ***F*** **-Test)**							
DLPFC	L	146	11.78		−54	32	−8
Superior medial gyrus	L	297	11.99		−12	59	21
Inferior parietal cortex	R	215	12.76		44	−68	23
Precuneus	L	257	10.67		−3	−51	39

Activation patterns resulted from a group-level GLM. ‘Average effect N1’: *T*-contrast (*T*>3.29) testing for general effects of N1-amplitudes as a covariate. ‘Main effect N1’: *F*-contrast (*F*>6.48) testing for this covariate taking into account any differences between crossmodally manipulated n-back conditions. Both contrasts were inclusively masked (*p*<.05, uncorrected) with the effects of interest of the four HRF regressors and thresholded at *p*<.05 MC-corrected, k>125. P-values are only reported if surviving voxel-level FWE correction.

When taking into account the cross-modal condition effect and testing for unsigned differences between any of the N1 peak amplitude regressors with an *F*-Test, activation patterns consisted of focal activations in the left DLPFC and superior medial gyrus, inferior parietal cortex and precuneus.

Neither P2 peak amplitudes, nor N1-P2 absolute peaks explained inter-individual subject variance above the set Monte-carlo corrected threshold of p<.05.

### Primary Auditory Cortex Region of Interest Analysis

The GLM analyzing averaged time-series of each WM condition BOLD response from a region-of-interest analysis of left AC (main effect ‘n-back’, Wald *χ^2^*(3) = 8.52, *p* = .04) revealed a significant increase in deactivation from the fixation condition to the different WM load conditions. Post-hoc tests showed significant deactivation increases between 0-back and 2-back (*t*(14) = 2.82, *p* = .014) and between 1-back and2-back (t()14) = 2.59, *p* = .21) only if uncorrected for multiple comparisons (Table 5).

**Table 5:** Region-of-interest analyses of primary auditory cortices (AC), averages of mean activation (SD in brackets), subject to a generalized linear model (GLM) testing for significant differences between conditions.

	Primary auditory cortex	
WM-condition	Left	Right
**Fixation**	−0.15 (0.19)	−0.13 (0.26)
**0-back**	−0.07 (0.20)	−0.16 (0.28)
**1-back**	−0.07 (0.25)	−0.26 (0.29)
**2-back**	−0.29 (0.33)	−0.48 (0.38)

This effect was replicated in right AC (Wald *χ^2^*(3) = 12.56, *p = *.006). Post-hoc t-tests tests indicated significantly higher deactivation in 2-back compared to fixation (*t*(14) = 4.68, *p*<.001), 2-back compared to 0-back (*t*(14) = 4.57, *p*<.001) and 2-back compared to 1-back (*t*(14) = 3.224, *p* = .006).

### Psychophysiological Interaction

**Figure 5:**
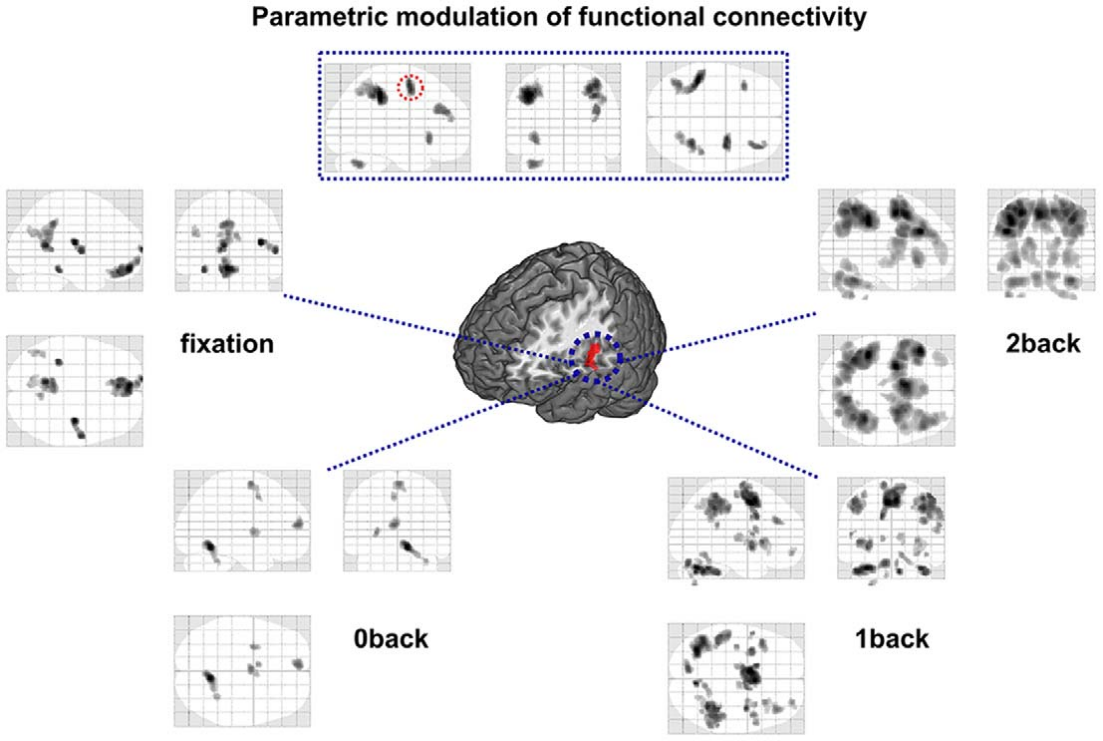
Functional connectivity of left auditory cortex (PPI seed region) in different visual WM-load conditions. The top display reflects the parametrically increasing functional connectivity with core regions from the visual WM-load (*p*<.05, MC-corrected, k>125). The red circled region survives inclusive masking with the initial GLM testing for a parametric WM.-load increase. Lower displays show task-dependent functional connectivity of left AC in different conditions. All contrasts resulted from a group-level random-effects GLM analyzing effects for PPI interaction parameters (task by seed region) (*p*<.05, FWE-corrected, k>20).

Testing task-related functional connectivity of left AC in each WM-condition yielded the following results: during fixation, the AC showed significant functional connectivity with the right primary AC, precuneus, fusiform gyrus, and ventromedial PFC (Table 6, Figure 5). This changed with increasing WM-load to a connectivity pattern increasingly representing the fronto-parietal WM network (SMA, DLPFC, inferior parietal lobe, thalamus, and cerebellum). The parametric effect of WM-load (T-contrast conjunction combining 2-back>1-back ∩ 1-back>0-back ∩ 0-back>fixation) revealed functional connectivity with bilateral inferior parietal lobes and sulci, several frontal areas (IFG, middle and superior frontal gyrus, SMA) as well as thalamus and lobules VI and VIIa crus I of the cerebellum (Table 6). When masking this contrast with the parametric effect of the initial nback GLM (inclusively masking, *p*<.05 uncorrected) we found one cluster in right DLPFC (MC-cluster-corrected, *p*<.05).

**Table 6:** Functional connectivity of left auditory cortex in different visual WM conditions.

Brain region	Hemisphere	Size	T	p*	x	y	z
**Parametric WM-load (conjunction 2-back>1-back ∩ 1-back>0-back ∩ 0-back>fixation)**							
Inferior frontal gyrus/DLPFC	L	183	4.46		−36	21	−5
Middle frontal gyrus	R	342	4.53	.032	39	41	26
	R	499	5.04		29	0	51
Inferior parietal gyrus	L	1085	5.57	<.001	−42	−38	44
	R	734	4.94	<.001	35	−45	44
Lobule VIIa crus I	L	288	4.54		−38	−62	−35
**Fixation**							
Orbitofrontal/rectal gyrus	L	1682	7.91	<.001	−5	53	−17
Medial frontal gyrus	L	83	7.22	<.001	−12	69	8
Heschl's gyrus	R	457	7.72	<.001	39	−15	15
Angular gyrus	L	208	6.22	.004	−45	−63	27
Precuneus	L	1502	7.43	<.001	−9	−54	12
	R	114	6.24	.004	8	−60	24
Fusiform gyrus	L	121	7.22	<.001	−33	−38	−17
**0-back**							
Medial frontal gyrus	L	298	7.04	<.001	−6	54	14
SMA	R	258	6.91	<.001	0	−5	62
	L	62	6.36	.003	−6	5	47
Putamen	L	130	6.65	<.001	−29	−2	3
Lobule VI	R	601	8.45	<.001	11	−56	−14
**1-back**							
Inferior frontal gyrus	L	294	7.14	<.001	−41	17	2
	V	112	6.79	.001	47	5	32
	R	135	6.52	.002	57	8	17
Middle frontal gyrus	R	214	7.01	<.001	41	35	27
	R	266	7.57	<.001	38	−3	54
Superior frontal gyrus	R	65	6.48	.002	32	54	−12
Rolandic Operculum	L	31	6.61	.001	−53	5	11
Precentral gyrus	L	68	6.50	.002	−38	−17	59
SMA/pCC	L	2369	8.53	<.001	−8	2	50
Inferior parietal lobule	L	954	7.40	<.001	−50	−36	50
	R	2504	7.72	<.001	39	−44	42
Insula	R	220	6.59	.001	32	20	3
Fusiform gyrus	R	29	6.22	.004	42	−59	−14
Putamen	L	276	7.35	<.001	−33	3	−5
	R	111	6.33	.003	35	8	−2
Thalamus	L	85	6.29	.003	−12	−14	6
	R	110	6.18	.005	9	−12	8
Lobule VIIb	L	37	6.11	.006	−8	−74	−39
Lobule VIIa crus I	L	1234	8.38	<.001	−33	−66	−30
Lobule VIIIa/VI	R	148	7.84	<.001	8	−68	−35
Lobule IV/VIIa	R	539	7.81	<.001	36	−51	−32
Lobule VI/V	R	124	6.53	.002	11	−56	−14
Lobule VI/VIIa crus I	L	284	7.39	<.001	−9	−77	−24
Lobule VIIb	L	51	6.45	.002	−27	−69	−51
**2-back**							
Inferior frontal gyrus	L	906	9.47	<.001	−36	21	−6
	R	1004	8.13	<.001	30	26	−3
DLPFC	L	6068	13.12	<.001	−48	26	29
	R	3246	9.80	<.001	45	30	33
Middle frontal gyrus	L	735	6.91	<.001	−39	48	12
	R	174	6.53	.002	33	53	−6
SMA	L	7299	12.58	<.001	−5	6	50
Inferior parietal gyrus	L	7784	13.17	<.001	−42	−39	45
	R	7855	11.84	<.001	36	−45	44
Fusiform gyrus	L	539	7.86	<.001	−53	−65	−15
	R	34	6.34	.003	44	−60	−14
Lentiform nucleus	L	1708	8.59	<.001	−18	−2	17
Thalamus	R	1246	8.01	<.001	18	0	17
Lobule VIIa crus I	L	3747	9.33	<.001	−36	−63	−33
Lobule VIIa crus I/VI	R	1333	1.41	<.001	36	−57	−32
Lobule VIIb	L	51	7.12	<.001	−27	−71	−51
Lobule VI/VIIIa	R	36	6.71	.001	39	−42	−53
Lobule VI	R	66	6.54	.001	18	−53	−24

Parametric WM-load conjunction contrast, *T*>3.24, *p*<.05, MC-cluster-corrected, k>125. Simple main effects of WM-load conditions, *T*s>5.45, *p*s<0.05, FWE-corrected, k>20.

The results of the PPI analysis of right AC task-related connectivity yielded similar results. Functional connectivity with the left AC under fixation condition appeared slightly weaker but the parametrically increasing functional connectivity with the fronto-parietal WM network was replicated.

## Discussion

The present study investigated how increasing visual WM-load (T1) affected secondary fundamental auditory processing (T2). EEG-fMRI enabled us to identify WM load activation representing the primary task manipulation as well as precision in the temporal domain to analyze neural action of simultaneously ongoing auditory processing.

### Crossmodal Augmentation Effects in Unattended Task Processing

**Figure 6:**
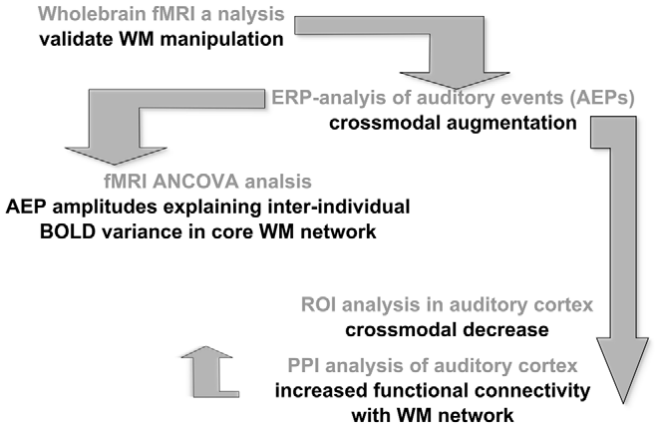
Outline of the reciprocal analysis strategy carried out with simultaneous EEG-fMRI, investigating differential effects of primary task visual working memory load on secondary task fundamental auditory processing. fMRI = functional magnetic resonance imaging, WM = working memory, ERP = event-related potential, ANCOVA = analysis of covariance, BOLD = blood oxygenation level dependent, ROI = region-of-interest, PPI = psychophysiological interaction.

The primary task manipulation, a visual WM task, was validated by the fMRI results showing bilateral fronto-parietal and subcortical neural activation patterns corresponding to WM-load [23] and participants' behavior (increasing RTs and decreasing hit rates with increasing WM load, Figure 6).

Basic auditory processing, represented by saturated standard tones revealed a simultaneously happening stepwise increase of the AEP corresponding to a gradual increase in crossmodal WM load. Cross-modal attention effects have been repeatedly reported on auditory processing [1], [8], [18]. Nevertheless, first, results are contradictory and range from an automaticity assumption regarding the basic analysis of auditory perception [6], [7], to proposing differential effects of the primary task on several outcome parameters of the secondary task [10], [11]. Secondly, to our knowledge, fundamental processing, as expressed by the N1-P2 complex, had not been explicitly studied in a comparable design.

Our results contradict the automaticity assumption and show a clear susceptibility of fundamental auditory processing to cross-modal WM load manipulation, systematically investigated by using four modulations. A susceptibility to crossmodal cognitive load influences supports and extends Haroush’s [12] findings. They argued that the enhanced processing of T2 was due to a lack of executive control which usually causes an attenuation of second-modality input. This lack was present because the system, being busy with T1 consolidation, was challenged to a point of cognitive overflow where the effective suppression of inputs other than the attended one could not be guaranteed anymore. While the attentional blink paradigm challenges participants to the limits of conscious perception via temporal manipulation of sensory perception [52], [53], [54], [55] and working memory consolidation in a short time period [12] the applied n-back task employed several levels of working memory load. Here, under low T1-load, the crossmodal processing of unattended T2-processing was smaller (smaller AEP amplitudes) than when compared to high T1-load (higher AEP amplitudes). Because an intuitive cause of this would have to be found in generators of this response, we carried out a region-of-interest analyses in the primary auditory cortices in which active suppression of secondary input is either not possible or not intended due to mechanisms of generalizing attentional resources as described next.

### Re-allocation of Cognitive Resources to the Primary Task and Spread of Attention

Potential effects of decreasing central executive control inhibition on secondary task processing were investigated with region-of-interest analyses of the BOLD response in both primary auditory cortices (ACs) as important auditory response generators [42], [43]. The results revealed AC deactivation associated with cross-modal increasing task demands in T1. This, at first, questioned the presence of break-down of cognitive control on primary sensory areas because the latter should have intuitively caused an *increase* of the (uninhibited) primary AC. Instead, this processing decrease of a secondary task supported 'gain theory’ assumptions of resource allocation to the primary attended modality [56], [57].

However, the auditory cortices are not the only generators of late AEPs [58] and attentional effects seem to play a crucial role here [17], [59]. This promotes the possibility of differential regional contributions to the AEP [60] when cognitive crossmodal load comes into play. Indeed, our data showed correlations of N1-amplitudes with BOLD activation in potential frontal contributors of AEPs [58]. When taking into account the cross-modal manipulation (main effect of covariate, *F*-contrast, Table 4), activation was present in DLPFC, superior medial gyrus and inferior parietal gyrus (Figure 4, yellow coloring). This hints to an involvement of the so-called ‘core’ network of WM-load activation [23] covarying with the electrophysiological response specifically when considering crossmodally manipulated WM load rather than if simply considering general effects the N1 amplitudes have on BOLD variance (green coloring).

Hence, the AEP seems to be associated with visual WM nodes. We further carried out a psychophysiological interaction analysis (PPI) which resulted in a parametric effect of task-dependent functional connectivity of AC with the WM network. This might be due to a stepwise re-allocation of cognitive resources to regions associated with processing primary cognitive load. Contrary, during fixation, where no cognitive load was imputed left AC connectivity patterns were present in its contralateral counterpart, precuneus and vmPFC.

We propose that low WM load did not intervene much with the limits of executive control and there was no necessity to re-allocate attentional resources. High load, however, subtracted attention from uni-modal sensory processing areas and allocated the available resources to the relevant neural structures which may contribute to a more increased joint generation of responses in the secondary modality [2], [14], [15], [61], [62]. This strongly suggests that the AEP is associated with nodes in a network, which may or may not biophysically contribute or modulate to its appearance.

Our findings may finally help to explain the often reported *decreases* of the auditory change-effects which is represented in a smaller difference between deviant and standard tone processing under high load. The current investigation of fundamental tone processing might be an important step in a sensitive approach for evaluating cognitive load effects on continuous stimulus processing in a different modality. It remains speculative if deviant processing which recruits automatic, bottom-up attentional resources [59,19 63] appears less sensitive to cognitive load manipulations compared to standard processing (the datasets included only 45 deviant tones in total and we refrained from a condition-wise analysis).

### Limitations and Conclusion

Using a simultaneous EEG-fMRI measurement protocol we demonstrated that basic auditory processing is systematically related to cross-modal cognitive load. We extend existing findings and show that increasing cognitive load impacts secondary task sensory processing. While a beneficial effect of crossmodal task load was found in elctrocortical responses of basic auditory processing by the vertex potential a deactivations of primary auditory cortices contradict a break down of executive control and rather points to a reallocation of attentional resources and spread of attention. However, the region-of-interest analysis was based on an anatomical template of the complete Heschl’s gyrus, which neglects to pay tribute to potential differential effects within this region.

A potential caveat that should be considered is the mixing of effects in the fMRI analyses (main and PPI) in which one block consisted of multiple letter presentations, but also tone presentations. While this should be generally considered, a condition-effect is likely to be caused by the manipulated letter presentation (n-back), however, an interaction with the (stable) tone presentation cannot be ruled out.

Another aspect refers to trial-by-trial fluctuations as they have been shown to be of predictive value in simultaneous EEG-fMRI designs [64]. While regarding auditory trial-by-trial coupling is a matter of ongoing debate [24], our design with an inter-trial interval of 1.4 s of the tones did not allow for an event-related investigation of the BOLD response because of its inertness.

Summarized, we could show that auditory cortices are increasingly connected to exactly those regions, which are up-regulated during increasing demands of cognitive/attentional control. We further demonstrate that cognitive load crossmodally manipulates auditory-cortex functional connectivity patterns via mechanisms of spread of attention. This causes a re-allocation of neural networks associated with the generation of a secondary sensory memory signal. To what extent the identified nodes actually represent neural generators of the AEP remains to be explicitly tested.
